# Comparison of the Active Compositions between Raw and Processed *Epimedium* from Different Species

**DOI:** 10.3390/molecules23071656

**Published:** 2018-07-07

**Authors:** Huamei Zhang, Hui Wang, Juan Wei, Xiaopeng Chen, Mengjie Sun, Huizi Ouyang, Jia Hao, Yanxu Chang, Zhiying Dou, Jun He

**Affiliations:** Tianjin State Key Laboratory of Modern Chinese Medicine, Tianjin University of Traditional Chinese Medicine, 312 Anshanxi Road, Nankai District, Tianjin 300193, China; zhanghm0527@163.com (H.Z.); tgwanghui@163.com (H.W.); 15222797261@163.com (J.W.); xpchen@tjutcm.edu.cn (X.C.); 15122865529@163.com (M.S.); huihui851025@163.com (H.O.); haojiatjtcm@126.com (J.H.); tcmcyx@126.com (Y.C.); zhiyingdou@163.com (Z.D.)

**Keywords:** *Epimedium*, identification, processing, LC–MS/MS, comparison

## Abstract

*Epimedium* herb is one of the most vital traditional Chinese medicines (TCMs), which is used for “nourishing the kidney and reinforcing the Yang”. In the guidance of TCM theory, *Epimedium* herb is usually processed with lamb oil to increase its efficacy. The contents of active ingredients in different *Epimedium* are significantly varied, which may derive from their different species, regions and processing methods. In this research, 13 batches of raw *Epimedium* collected from 6 provinces were identified. After optimization of the processing method of *Epimedium*, a liquid chromatography–mass spectrometry (LC–MS/MS) method for simultaneous determination of 16 compounds was established to evaluate the quality of raw and processed. Then the multivariate statistical technique was applied to compare different batches of *Epimedium* based on the LC–MS/MS data. As a conclusion, the herbs collected from 6 areas were ascribed to 5 species by microscopic and appearance features. Meanwhile, all of the raw and processed samples were classified by partial least squares discriminant analysis (PLS-DA) based on the 16 analyzed compounds. The comparison results indicate that processing and species both have important influences on *Epimedium* compositions contents.

## 1. Introduction

*Epimedium* herb, commonly named as Yinyanghuo in Chinese, has its origins in the Berberidaceae family which include *Epimedium koreanum* Nakai, *Epimedium brevicornum* Maxim., *Epimedium pubescens* Maxim., *Epimedium sagittatum* (Sieb.et Zucc.) Maxim. and *Epimedium wushanense* T. S. Ying [1]. According to the “Shen Nong’s Herbal Classic” records, as one of the tonic TCMs, *Epimedium* herb has been used for more than 2000 years [2].

Chemical studies reveal that more than 260 ingredients have been isolated from *Epimedium*, such as flavonoids, polysaccharides, essential oils, phytosterols, phenolic acids and alkaloid [3,4]. Pharmacological studies demonstrate that ingredients in *Epimedium* possess various attributes. For example, flavonoids, the major constituents in *Epimedium* which including baohuoside II, baohuoside I, epimedin A, epimedin B, epimedin C, quercitrin, sagittatoside A, sagittatoside B, hyperoside, icariside I, icariin and astragalin, have been widely used for improving cardiovascular and promoting sexual function [5,6]. They also show anti-osteoporosis, anti-inflammation, anti-cancer and anti-oxidation effects [7,8,9,10,11]. Meanwhile, the phenolic acids such as chlorogenic acid, neochlorogenic acid and cryptochlorogenic acid have extensive pharmacological actions including anti-inflammation, anti-fatigue and antitumor properties [12,13,14]. Furthermore, alkaloid such as magnoflorine is found to possess anti-glycemic and anti-oxidant activities [15,16].

In TCM theory, processing is a necessary procedure, which can enhance clinical efficacy and decrease the toxicity of herbs [17]. For *Epimedium* herb, much research suggests that processed and unprocessed herbs have a different effect in pharmacological and clinical. The drug processed has greater efficacy for improving sexual desire and performance, while the raw has stronger efficacy in osteoporosis and rheumatism [18,19]. Modern studies focusing on pharmacological properties reveal that the processed method is an important factor influencing composition contents. Chen et al. reported that the contents of flavonoids were increased when the processing temperature was 60 °C [20] while Gao et al. claimed that 120 °C may be the best [21]. Comparing the contents of flavonoids after processing, Li et al. pointed out roasting was better than frying [22]. In practical production, there are many processing methods for Chinese herbs, including stir-frying, steaming, calcining, roasting and boiling. The Chinese Pharmacopoeia (2015 edition) record stir-frying with lamb oil as the processing method of *Epimedium* herb but not in detail [1]. Thus, it is necessary to optimize the traditional processing method to gain standardization.

The previous study reported that both species and region had a notable impact on the contents of TCMs [17]. Gao et al. indicated that the magnolia content of *E. koreanum* Nakai was 10 times higher than *E. brevicornu* Maxim. Moreover, the *E. brevicornum* Maxim. collected from Gansu province were better than other provinces [23]. So it is important to develop a reliable method for *Epimedium* identification based on the appearance and microscopic features.

In general, baohuoside I and icariin are considered as the major active components and the quality marker of Epimedium herb [1]. However, other ingredients such as phenolic acids and alkaloid with verified pharmacological activities are also abundant in *Epimedium*. Comprehensive quality control demands a method to analyze multiple active components of *Epimedium* simultaneously. Chen et al. compared the methods for determination of 15 flavonoids in raw *Epimedium* by high-performance liquid chromatography (HPLC) and ultra performance liquid chromatography (UPLC). The analysis time of the UPLC method was shorter than HPLC, and the UPLC method was more sensitive [24]. Moreover, Zhu et al. had established the fingerprint chromatogram of *Epimedium* by UPLC, and found there were 5 differences in fingerprint between crude and processed product [25]. Naseer’s research demonstrated that the HPLC–MS method exhibits higher sensitivity, lower LOQs and shorter analysis time compared with the HPLC–ultraviolet (UV) and UPLC–UV methods [26,27].

Considering all these factors, *Epimedium* from 6 regions in China were ascribed to 5 species (*E. koreanum* Nakai, *E. brevicornum* Maxim., *E. pubescens* Maxim., *E. sagittatum* (Sieb.et Zucc.) Maxim. and *E. wushanense* T. S. Ying) by appearance and microscopic features. Then, 13 batches of samples were processed by optimized method and 16 ingredients including 12 flavonoids such as baohuoside II, baohuoside I, epimedin A, epimedin B, epimedin C, quercitrin, sagittatoside A, sagittatoside B, hyperoside, icariside I, icariin and astragalin, 3 phenolic acids such as chlorogenic acid, neochlorogenic acid, cryptochlorogenic acid and 1 alkaloid such as magnoflorine were simultaneously evaluated by LC–MS/MS. The present assay was further applied to investigate and compare the main components of crude and processed *Epimedium* using multivariate data analysis (partial least squares discriminant analysis (PLS-DA)) [28,29].

## 2. Results and **Discussion**

### 2.1. Appearance and Microscopic Features of Epimedium

As shown in Figure 1, it is clear that the different features appear in leaf size, shape, material and texture. It is also obvious that microscopic features differ in leaf upper epidermis, leaf lower epidermis, fibers, non-glandular hairs, glandular hairs, reddish-brown content and oil cells. The detail information and identification of *Epimedium* were demonstrated in Table 1. The appearance and microscopic features of *Epimedium* are shown in Figures S1–S13.

For TCM, morphologic analysis, microscopy analysis and DNA analysis were useful tools for taxonomic identification of plant species. DNA barcoding and microsatellite analyses to distinguish plant species were more accurate [30,31,32,33,34]. In the present paper, considering that they are effective and easily undertaken, morphologic and microscopy analysis were chosen as the reasonable method for herb identification. In the future, it is necessary to identify herbs comprehensively by various methods.

### 2.2. Optimization of Processing Conditions

The Chinese Pharmacopoeia indicates that icariin is the quality indicator for raw *Epimedium* herb, while icariin and baohuoside I for processed *Epimedium* herb. Flavonoids are considered as the major effective compounds in *Epimedium*, meanwhile glycosyl in the structure of flavonoids could be removed, such as epimedin C could transfer into icariin. Therefore, to optimize processing conditions, flavonoids including epimedin A, epimedin B, epimedin C, icariin and baohuoside I were chosen as the evaluation indicator. In order to obtain the optimal processing method, the processing heat (200 w, 400 w and 600 w), processing time (5 min, 10 min and 15 min) and samples weight (5 g, 10 g and 15 g) were selected as main elements. As shown in Table 2, the maximum content of total flavonoids could be achieved when 15 g of raw samples is heated under 200 w for 5 min. As a result, the method was selected as the optimization method to further process raw *Epimedium*.

The content of total flavonoids decreases with an increase in the processing time from 5 min to 15 min. At the same time, the content increases when the processing heat decreases from 600 w to 200 w. The reason for the content change after processing is high temperature destroys the structure of unstable constituents. For example, the sugar moieties of the glycosides are usually rhamnose, glucose, xylose or their corresponding acetyl or coumaroyl sugars at C-3 and/or C-7 positions may be lost. Regarding processing, the impact of the various factors is as follows: processing heat > processing time > samples weight.

### 2.3. Quantification the Raw and Processed Samples by LC–MS/MS

Causes of differences in pharmacological and clinical effects between processed or unprocessed Epimedium herb are still indeterminate. To find the material basis of processing, LC–MS/MS was applied to detect multiple components of *Epimedium.*

#### 2.3.1. Method Validation

**Figure 2:**
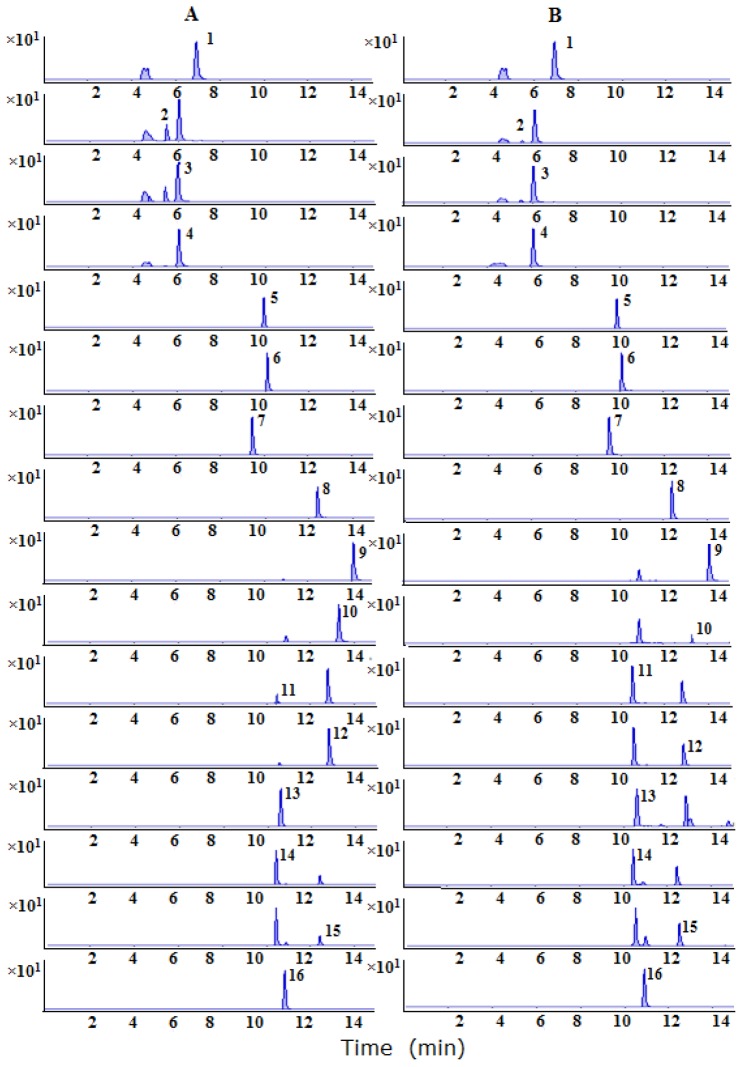
Multiple reaction monitoring (MRM) chromatograms of magnoflorine (1); neochlorogenic acid (2); chlorogenic acid (3); cryptochlorogenic acid (4); astragalinbaohuoside (5); quercitrin (6); hyperoside (7); baohuoside II (8); baohuoside I (9); icariside I (10); epimedin B (11); sagittatoside B (12); epimedin C (13); epimedin A (14); sagittatoside A (15); icariin (16); (**A**) standard solution; (**B**) *Epimedium* sample.

The linear calibration curves of peak areas (y) vs. concentrations (x) were plotted for 16 compositions. The regression coefficients (r) are >0.996 for the 16 compounds, indicating a good linearity within a relatively wide range of concentrations. The lower limits of quantitation (LLOQs) are all less than 1 ng/mL for all compositions. For the precision, the relative standard deviations (RSDs) for relative contents of 16 characteristic components are <3.53%, respectively. In repeatability test, RSD values for relative contents range from 1.02% to 9.79%. The results indicate that the current method has a satisfactory precision and repeatability. The stability presented as RSD is in the range from 2.99% to 9.27%, indicating that the samples are stable within 24 h. The recoveries of six replicates range from 90.32% to 112.40% for the 16 analytes. The results indicate that the efficiency of sample preparation is acceptable in the current condition. The typical multiple reaction monitoring (MRM) chromatograms of the analytes were shown in Figure 2. The validation data showed in Table 3 are considered to be satisfactory for subsequent analysis of all of the samples. The data of the present LC–MS/MS method demonstrate that it exhibits higher sensitivity, lower LLOQs and shorter analysis time compared with the existing HPLC-UV and HPLC–MS methods [24,25,26,27].

#### 2.3.2. Quantitative Analysis of Raw and Processed Products

**Figure 3:**
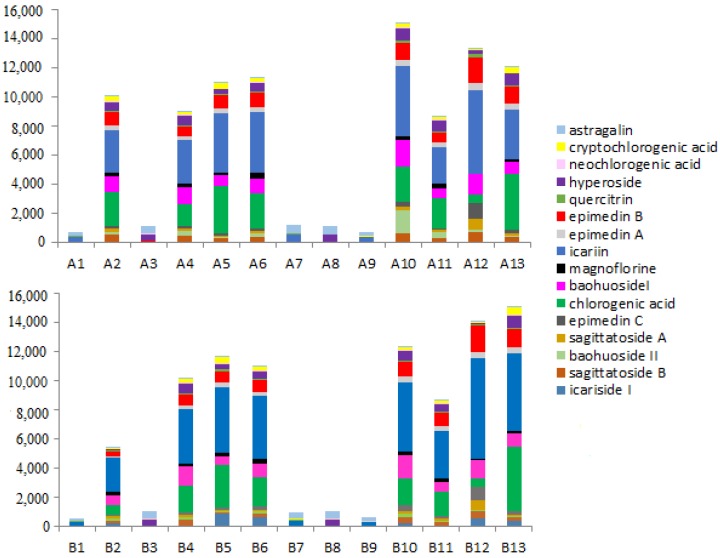
Contents of 16 components in different batches of raw and processed *Epimedium* (μg/g): (A) raw; (B) processed. The sample numbers were same as in Table 1.

The validated method was applied to the analysis of 13 batches of raw and processed *Epimedium* samples. A total of 16 active ingredients were quantified with the external standard method based on their respective calibration curves. The contents of 16 compounds in raw and processed *Epimedium* samples were listed in Table 4. As shown in Figure 3, there are changes in contents of analytes, but not in the composition between raw and processed samples.

#### 2.3.3. The Results of Data Analysis

**Figure 4:**
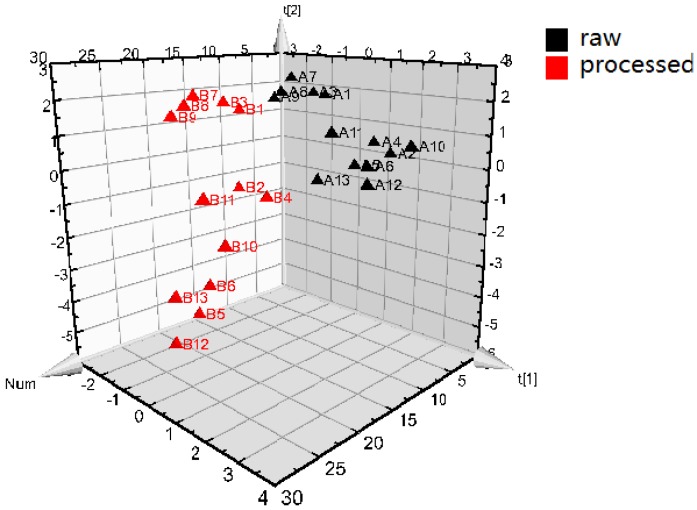
Partial least squares discriminant analysis (PLS-DA) 3D score scatter plot for raw (A) and processed (B) *Epimedium* (R2X = 0.656, R2Y = 0.418, Q2 = 0.081).

The LC–MS/MS results were further analyzed by PLS-DA. In Figure 4, the three-dimensional (3D) score plot of PLS-DA was carried out to measure the difference between raw and processed *Epimedium*. All samples of raw (A1–A13) clusters are in a small region, which are distinguished from the processed samples. The processed samples (B1–B13) are clustered in another relatively discrete larger sphere, which indicates the qualities of the raw samples are more stable than the processed samples. Constituents with large loading values can be considered as markers, which contribute obviously to the classification of the samples. In the present study, the potential active ingredients whose VIP > 1 are icariside I, baohuoside II, quercitrin, icariin, and sagittatoside B. The raw and processed samples could be distinguished clearly, which explain that processing plays a crucial role in the contents change of *Epimedium*. The quality markers of processed *Epimedium* are always chosen as epimedin A, epimedin B, epimedin C, icariin and baohuoside I [35]; however, the present research has found the ingredients affected most by processing are icariside I, baohuoside II, quercitrin, icariin, and sagittatoside B. Therefore, the study provides new point of view on indicators to the further optimize processing conditions of *Epimedium*.

**Figure 5:**
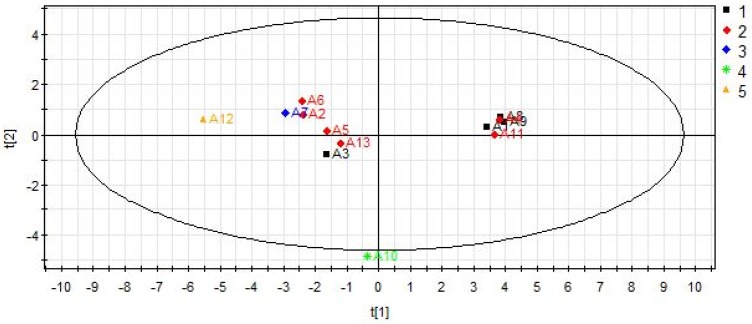
PLS-DA score scatter plot for samples collected from different species: (1) *E. sagittatum* (Sieb.et Zucc.) Maxim.; (2) *E. koreanum* Nakai; (3) *E. wushanense* T. S. Ying; (4) *E. brevicornu* Maxim.; (5) *E. pubescens* Maxim. The denotations from numbers 1 to 13 were the corresponding sample numbers as listed in Table 1 (R2X = 0.974, R2Y = 0.971, Q2 = 0.259).

As shown in Figure 5, most samples are clearly clustered into distinct groups corresponding to species. Distinguishing from other samples, all samples of *E. koreanum* Nakai (A2, A4, A5, A6, A11 and A13) cluster in one region. *E. pubescens* Maxim. (A12) is in the left quadrant, while *E. sagittatum* (Sieb.et Zucc.) Maxim. (A1, A3, A8, A9) is in the right quadrant. *E. wushanense* T. S. Ying (A7) is close to *E. pubescens* Maxim. But *E. brevicornu* Maxim. (A10) is away from all other samples. The corresponding scores plot combined with VIP values screen out active ingredients for the differentiation of varieties including baohuoside II, astragalin, neochlorogenic acid, magnoflorine, quercitrin and hyperoside. The important variables are selected as VIP > 1. The results of PLS-DA demonstrate variations in the chemical content of *Epimedium* from different species.

**Figure 6:**
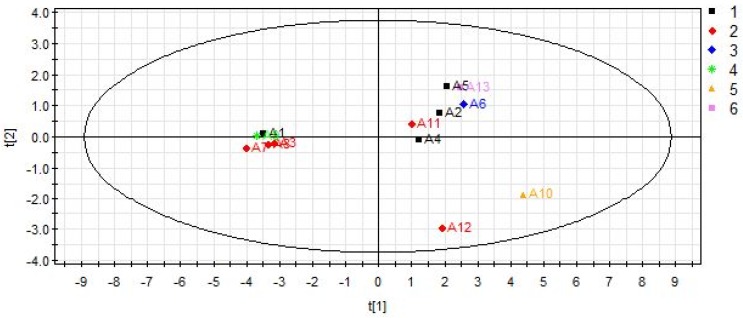
PLS-DA score scatter plot for samples collected from different areas in China. (1) Jilin; (2) Gansu; (3) Shan’anxi; (4) Sichuan; (5) Hubei; (6) Liaoning. The sample numbers in the plot were in accordance with the batch numbers in Table 1 (R2X = 0.761, R2Y = 0.220, Q2 = 0.135).

As shown in Figure 6, the plot was used to assess the difference in samples collected from various regions. PLS-DA results reveal that six provinces of samples could not be distinguished clearly. The samples (A1, A2, A4 and A5) obtained from Jilin appear close to Shan’anxi, Gansu and Liaoning provinces. Batches of samples 3, 7, 8, 11 and 12 all from Gansu province are dispersed in both negative and positive axis. The figure reveals that the factor of the region has no significant impacts on the classification of *Epimedium*.

## 3. Materials and Methods

### 3.1. Plant Material

In the present study, 13 batches of *Epimedium* were collected from different regions of China during 2015 and 2016. The origins of the samples were shown in Table 1.

### 3.2. Chemicals

Acetonitrile, methanol (Thermo Fisher Scientific, Waltham, MA, USA), formic acid (ROE SCIENTIFIC INC Newark, NJ, USA) were all of HPLC grade. Analytical grade ethanol was obtained from Tianjin Guangfu Science Co. Ltd. (Tianjin, China). Ultrapure water was prepared by a Milli-Q water purification system (Millipore, Milford, MA, USA). The reference standards (purity > 98%) of baohuoside II, baohuoside I, epimedin A, epimedin B, epimedin C, quercitrin, sagittatoside A, sagittatoside B, hyperoside, chlorogenic acid, magnoflorine, neochlorogenic acid, cryptochlorogenic acid, icariside I, icariin and astragalin were purchased from Tianjin, Yifangzhongkang Pharmaceutical Technology Co. Ltd. (Tianjin, China). Lamb oil was purchased from Yuquan Road Market (Tianjin, China).

### 3.3. Identification of Epimedium

The species of *Epimedium* were identified according to the leaves’ appearance and microscopic features. After screening with the No. 4 sieve, each dried sample was ground to powder by an electric grinder. Samples were placed on the slides and permeated twice with 1–3 drops of chloral hydrate, and then sealed by diluted glycerol and coverslip. For observation, *Epimedium* powder samples were taken with a digital camera and light microscope with 20 times magnification.

### 3.4. Optimization of the Processing Method of Epimedium

Lamb oil, 20% weight of raw samples, was melted in a hot pot. Raw *Epimedium* was then added and heated with constant tossing or stirring until the raw samples became sheeny and yellow. In order to optimize the processing method of *Epimedium*, the effects of processing technological factors, including processing heat, processing time and weight of *Epimedium* were investigated by orthogonal experimental design (L9 (3) ^4^). Total flavonoids (epimedin A, epimedin B, epimedin C, icariin and baohuoside I), as major active constituents, were chosen as the marker chemicals to evaluate the quality of processed *Epimedium* (Table 5).

### 3.5. Quantification by Liquid Chromatography–Mass Spectrometry (LC–MS/MS)

#### 3.5.1. Preparation of Samples

Thirteen batches of *Epimedium* were processed by the optimized method. Then, 100 mg *Epimedium* samples (raw or processed) were extracted with 100 mL of 70% aqueous ethanol under ultrasonic extraction conditions over 60 min at room temperature. After filtering through a nylon membrane filter (0.22 μm), the filtrate was used as the test solution.

Sixteen reference standards (magnoflorine, chlorogenic acid, cryptochlorogenic acid, neochlorogenic acid, hyperoside, epimedin A, epimedin C, sagittatoside B, baohuoside I, icariin, astragalin, quercitrin, baohuoside II, icariside I, epimedin B and sagittatoside A) were dissolved in methanol at a final concentration of 1 mg/mL as stock solutions, respectively. Working standard solutions were further obtained by appropriate stock standard solutions mixed and diluted with methanol.

#### 3.5.2. Chromatographic and Mass Spectrometry (MS) Conditions

Chromatography was performed on a CORTECSR C18 column (150 × 4.6 mm, 3 μm) at a 0.3 mL/min flow rate. The separation was obtained using the following gradient program: 0–4 min, 20–50% B; 4–5 min, 50–60% B; 5–15 min, 60–70% B (A: water containing 0.1% formic acid, B: acetonitrile). The injection volume was 5 µL and the column temperature was set at 30 °C.

The optimized conditions for MS detector were as follows: capillary voltage, −4000 V; drying gas (N2) flow rate, 9.0 L/min with a temperature at 300 °C; nebulizer pressure, 20 psi. The MRM scanning mode was employed for quantification in negative mode simultaneously. The mass spectra properties of 16 analytes were shown in Table 6. Data analysis was performed using Masshunter Workstation Software from Agilent Technologies (version B.04.00).

#### 3.5.3. LC–MS/MS Method Validation

The linearity of the assay for the test compounds was performed by least-square linear regression of 16 analytes-to-standard peak area ratios (y) versus the normalized standard concentration (x). The LLOQ for each sample was defined by the concentrations that generated peaks with signal-to-noise values (S/N) of 5. For precision, the method was evaluated by intraday and interday variability. The RSDs were calculated as the measure of precision. In the repeatability examination, six replicates of the samples from the same batch were extracted and analyzed. To evaluate the stability of analytes, sample solutions were stored at room temperature and then analyzed by replicate injection at 0, 2, 4, 8, 12 and 24 h, the RSDs were used to assess the stability. The recovery was evaluated by adding the standard solution to samples, which was used to further investigate the accuracy of the method. In the study, a known amount of 16 standards were added to *Epimedium* samples in 100 mL of 70% aqueous ethanol. The samples were thoroughly mixed before analyzing by LC–MS/MS. The recoveries were calculated by the formulae: recovery (%) = (amount found − original amount)/amount spiked × 100%.

### 3.6. Statistical Analysis

The differences of *Epimedium* were analyzed by PLS-DA. The method established the regression relationship between the matrixes, so as to get a better regression prediction result. When the supervised pattern recognition method was employed, the samples would divide into training and validation set. The classification model is obtained by training set, and the established model is used to predict the validation set. In the research, the validated method was applied to analyze *Epimedium* samples, including 13 batches of raw *Epimedium* samples (A1–A13) and 13 batches of processed *Epimedium* samples (B1–B13). A total of 16 compounds, including 12 flavonoids (hyperoside, epimedin A, epimedin C, sagittatoside B, baohuoside I, icariin, astragalin, quercitrin, baohuoside II, icariside I, epimedin B and sagittatoside A), 3 phenolic acids (chlorogenic acid, cryptochlorogenic acid, neochlorogenic acid) and 1 alkaloid (magnoflorine) were used to evaluate the changes of *Epimedium* after processing. The statistical performances of the models were evaluated by R2X, R2Y, and Q2. Statistical analysis was analyzed by SIMCA-P 12.0 software (Umetrics, Umea, Sweden).

## 4. Conclusions

At the beginning of the research, the assay was performed to exam Epimedium herb based on the microscopic features and appearance, which has become a specific and effective tool for herbal medicine identification. Then, research was carried out on the optimization of processing conditions. In addition, the contents of 26 batches *Epimedium* from different species, varied regions and different processing methods were investigated by LC–MS/MS, which enables more accurate monitoring and control of the herb quality. The LC–MS/MS quantification data of 16 active compounds were analyzed by PLS-DA. The PLS-DA results indicate that *Epimedium* is significantly different, in relation to the factors of the species as well as the processing method. Furthermore, an important finding is that six provinces of samples could not be classified into six sub-clusters by PLS-DA, which means the contents of 16 active compounds in *Epimedium* have no strong relation with the factor of the region.

## Figures and Tables

**Figure 1:**
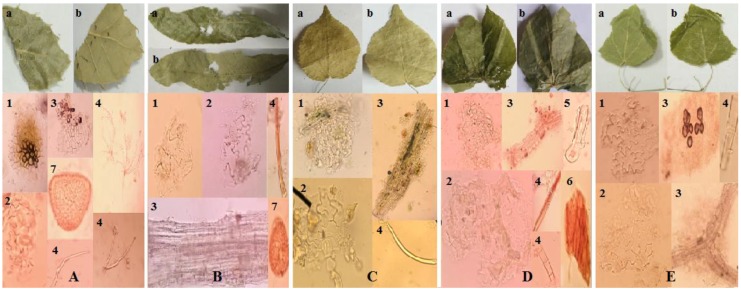
The appearance and microscopic features of *Epimedium* samples: (**A**) *E. sagittatum* (Sieb.et Zucc.) Maxim.; (**B**) *E. koreanum* Nakai; (**C**) *E. wushanense* T. S. Ying; (**D**) *E. brevicornu* Maxim.; (**E**) *E. pubescens* Maxim.; (a) leaf upper surface; (b) leaf lower surface; (1) leaf upper epidermis; (2) leaf lower epidermis; (3) fibers; (4) non-glandular hairs; (5) glandular hairs; (6) reddish-brown content; (7) oil cells.

**Table 1:** Detailed information of 13 batches *Epimedium*.

Batch	Origin	Appearance Features	Power Characteristics	Identification
1	Jilin	Leaves thick, leathery.	Lower and upper epidermal cells slightly wavy, relatively small; stomas dense; with verrucae on the surface.	*E. sagittatum* (Sieb.et Zucc.) Maxim.
2	Jilin	Leaves thin, relatively large, elliptical or subrounded in shape, margin serrate.	Epidermal cells slightly wavy, lower epidermal stomas and non-glandular hairs on them; non-glandular hairs straight or slight wavy, few undulant curvy; cells very big, some cells containing reddish-brown content.	*E. koreanum* Nakai
3	Gansu	Leaves leathery, oblong or lanceolate in shape.	Lower and upper epidermal cells slightly wavy, relatively small; with verrucae on the surface.	*E. sagittatum* (Sieb.et Zucc.) Maxim.
4	Jilin	Leaves thin, relatively large.	Epidermal cells slightly wavy, lower epidermal stomas and non-glandular hairs on them; non-glandular hairs straight or slightly wavy, few undulant curvy; cells very big, some cells containing reddish-brown content.	*E. koreanum* Nakai
5	Jilin	Leaves subrounded, apical leaf relative long, with serrate on the margin, base deep heart-shaped and askew, leaflets slightly large, leaves thin, membranous.	Epidermal cells slightly wavy, lower epidermal stomas and non-glandular hairs on them; non-glandular hairs straight or slightly wavy, some cells containing reddish-brown content.	*E. koreanum* Nakai
6	Shan’anxi	Leaves elliptical or subrounded, apical leaf relative long, with serrate on the margin, base deep heart-shaped and askew, leaflets slightly large, leaves thin, membranous.	Epidermal cells slightly wavy, lower epidermal stomas and non-glandular hairs on them; non-glandular hairs straight or slightly wavy, few undulant curvy; cells very big, some cells containing reddish-brown content.	*E. koreanum* Nakai
7	Gansu	Leaves lanceolate, apical leaf relative long, with serrate on the margin.	Epidermal cells slightlv wavy; few non-glandular on them, cells straight or curvy, with few columns of calcium oxalate.	E. wushanense T. S. Ying
8	Gansu	Leaves leathery, oblong or lanceolate in shape.	Lower and upper epidermal cells slightly wavy, relatively small; stomas dense; with verrucae on the surface; columns of calcium oxalate scattered throughout.	*E sagittatum* (Sieb.et Zucc.) Maxim.
9	Sichuan	Leaves thick, leathery, base askew.	Lower and upper epidermal cells slightly wavy, relative small; stomas dense; columns of calcium oxalate scattered throughout.	*E. sagittatum* (Sieb.et Zucc.) Maxim.
10	Hubei	Leaflets in base deep heart-shaped, with serrate on the margin, leathery.	Epidermal cells slightly wavy; anomocytic stomata; non-glandular hairs relative less; columns of calcium oxalate scattered throughout; some cells containing reddish-brown content.	*E.brevicornu* Maxim.
11	Gansu	Leaves thin, relatively large.	Epidermal cells slightly wavy, lower epidermal stomas and non-glandular hairs on them; non-glandular hairs straight or slightly wavy, few undulant curvy; cells very big, some cells containing reddish-brown content.	*E. koreanum* Nakai
12	Gansu	The lower surface of leaf and petiole densely covered with villous pilose, leaves thin, leathery.	Non-glandular slightly fine and wavy; glandular rare; upper and lower epidermal cells curved or irregular; anomocytic or anisocytic stomata sparse; fibers visible; some containing secretions.	*E. pubescens* Maxim.
13	Liaoning	Leaves elliptical or subrounded, apical leaf relative long, with serrate on the margin, base deep heart-shaped and askew, leaflets slightly large.	Epidermal cells slightly wavy, lower epidermal stomas and non-glandular hairs on them; non-glandular hairs straight or slightly wavy, few undulant curvy; cells very big, some cells containing reddish-brown content.	*E. koreanum* Nakai

**Table 2:** Orthogonal array design matrix L9 (3) ^4^ and experimental results (*n* = 3).

Test Number	Factors								
	Processing Time (min)	Processing Power (w)	Weight (g)	Epimedin A (mg/g)	Epimedin B (mg/g)	Epimedin C (mg/g)	Icariin (mg/g)	Baohuoside I (mg/g)	Total Yield (mg/g)
1	5	200	5	2.13	2.99	11.21	10.12	1.52	27.97
2	5	400	10	1.70	2.51	8.44	8.25	1.37	22.26
3	5	600	15	1.61	2.16	8.23	10.28	1.63	23.92
4	10	200	10	1.63	2.28	8.57	7.58	1.39	21.45
5	10	400	15	1.56	1.96	8.20	9.17	1.56	22.45
6	10	600	5	0.04	0.10	0.52	0.46	0.38	1.51
7	15	200	15	1.70	2.27	8.54	8.43	1.39	22.33
8	15	400	5	1.50	1.78	6.73	10.76	1.69	22.47
9	15	600	10	0.00	0.00	0.10	0.04	0.06	0.20

**Table 3:** Regression equation, linear range, correlation coefficients (r), lower limit of quantitation (LLOQ), precision, repeatability, stability, and recovery of 16 investigated analytes (*n* = 6).

Compunds	Regression Equation	Linear Range (ng/mL)	r	LLOQ (ng/mL)	Precision Relative Standard Deviations (RSD) (%)	Repeatability RSD (%)	Stability RSD (%)	Recovery (%)
Magnoflorine	y = 19.9023x + 10.0977	5–50,000	0.998	1.0	1.16	7.85	8.95	96.50%
Neochlorogenic acid	y = 38.3582x + 1.3968	5–50,000	0.998	1.0	2.92	1.60	8.36	94.67%
Chlorogenic acid	y = 133.5534x + 253.0658	5–50,000	0.999	1.0	3.53	1.02	8.60	112.40%
Cryptochlorogenic acid	y = 28.6432x − 1.0442	5–50,000	0.998	1.0	2.89	2.89	6.01	93.10%
Astragalin	y = 76.2325x + 45.2283	1–10,000	0.998	1.0	1.02	6.68	6.53	93.69%
Quercitrin	y =112.5154x + 21.2939	1–10,000	0.999	1.0	0.57	9.79	4.15	91.36%
Hyperoside	y = 85.7392x + 48.9969	5–50,000	0.997	1.0	0.65	3.76	2.99	100.45%
Baohuoside II	y = 180.1430x + 21.0798	1–10,000	0.997	0.5	0.82	7.94	4.88	90.32%
Baohuoside I	y = 165.3076x + 168.5352	5–50,000	0.999	0.5	0.29	2.62	4.76	92.43%
Icariside I	y = 236.7256x + 218.9144	1–10,000	0.996	1.0	0.86	8.22	9.27	108.30%
Epimedin B	y = 57.2633 + 3.9729	1–10,000	0.998	1.0	1.65	1.86	3.85	91.22%
Sagittatoside B	y = 88.9303x − 19.0810	5–50,000	0.999	0.5	0.91	3.66	4.01	100.18%
Epimedin C	y = 19.0452x − 2.5194	5–50,000	0.997	1.0	2.09	3.19	3.95	110.35%
Epimedin A	y = 25.9472x + 6.5190	5–50,000	0.999	1.0	1.94	2.30	6.21	105.60%
Sagittatoside A	y = 68.2445x +20.1904	1–10,000	0.998	0.5	0.59	3.77	5.89	94.98%
Icariin	y = 133.1104x + 838.2982	5–50,000	0.996	0.5	1.35	1.14	4.32	98.43%

**Table 4:** The contents of 16 compounds in *Epimedium* samples (μg/g).

Compunds	Sort	1	2	3	4	5	6	7	8	9	10	11	12	13
Baohuoside II	crude	0.98	101.19	0.69	183.95	49.40	132.40	0.35	0.38	0.31	882.15	248.12	78.82	41.46
	processed	0.53	120.28	0.67	103.19	35.77	102.85	0.35	0.23	0.26	179.68	44.06	61.73	39.82
Baohuoside I	crude	2.44	626.16	4.82	723.63	420.10	591.28	5.14	1.72	1.79	1125.14	411.42	820.44	484.79
	processed	2.54	392.24	5.37	774.33	366.89	573.90	6.40	3.32	2.89	945.51	432.14	760.07	553.46
Epimedin A	crude	0.33	1331.78	2.39	917.99	1323.22	1300.17	4.53	3.22	1.87	1697.70	1011.92	2054.51	1704.68
	processed	3.19	425.84	3.09	976.37	1124.78	1052.93	6.69	4.89	3.46	1422.28	1300.54	1807.19	1758.32
Epimedin B	crude	2.30	1668.05	4.64	1206.51	1496.90	1712.98	5.66	5.25	7.21	2065.07	1215.85	3015.30	1946.71
	processed	17.88	621.59	3.24	1312.94	1426.51	1417.14	6.26	7.29	3.91	1728.83	1611.52	3075.18	2089.55
Epimedin C	crude	2.69	977.79	3.87	1007.21	855.22	1148.50	8.35	5.50	5.14	1852.39	760.55	5328.93	1094.29
	processed	10.44	482.20	9.55	1080.77	792.09	979.93	5.86	4.35	9.70	1894.00	1078.07	4955.04	1100.49
Quercitrin	crude	9.42	79.08	10.40	80.74	127.82	101.90	10.26	10.87	19.22	89.37	86.85	216.49	69.50
	processed	11.86	35.24	9.80	63.92	133.40	85.15	8.39	9.35	8.13	69.50	48.78	101.32	58.07
Sagittatoside A	crude	0.64	326.58	0.53	262.79	128.74	226.19	0.54	0.63	0.48	403.78	175.85	1163.67	221.25
	processed	0.38	126.77	0.53	247.78	100.44	190.45	0.26	0.81	0.41	303.49	176.40	953.55	149.32
Sagittatoside B	crude	1.06	565.49	1.01	456.68	224.44	373.74	1.45	1.14	1.24	599.40	282.31	750.66	404.40
	processed	1.22	188.59	1.46	405.13	165.77	293.87	1.39	1.72	1.91	471.76	309.80	528.11	258.03
Hyperoside	crude	379.84	684.99	539.24	835.24	306.45	655.84	544.31	497.95	345.51	1011.95	870.43	271.50	952.46
	processed	294.44	107.31	452.53	784.14	387.38	553.44	425.78	485.90	362.86	829.46	608.12	119.50	1001.10
Chlorogenic acid	crude	12.54	1755.87	15.62	1110.14	2471.91	1799.81	14.11	9.09	6.72	1767.93	1542.55	475.88	2890.56
	processed	12.67	538.59	11.56	1371.74	2188.57	1520.72	18.48	14.32	12.41	1380.94	1218.47	384.22	3336.53
Magnoflorine	crude	-	1287.09	0.86	1141.12	1129.52	2291.76	0.41	0.66	-	1321.29	1335.76	350.53	939.18
	processed	-	1310.17	0.72	694.15	1320.46	1380.92	1.09	1.66	2.60	1249.40	1204.44	426.97	663.15
Neochlorogenic acid	crude	130.19	161.18	172.34	139.36	163.03	185.52	182.74	152.31	97.77	148.66	102.01	28.42	109.09
	processed	100.80	44.70	146.18	160.19	180.31	192.16	142.38	166.78	92.54	134.69	135.78	40.56	152.02
Cryptochlorogenic acid	crude	10.03	1020.15	14.48	627.72	1368.76	977.91	10.93	11.84	11.37	989.72	767.35	150.61	1567.19
	processed	9.04	242.51	11.48	795.01	1342.77	863.51	14.87	15.72	13.55	768.96	623.72	146.62	1856.38
Icariside I	crude	4.20	5.87	1.79	7.56	18.92	12.73	0.36	0.19	-	19.49	5.10	17.41	11.41
	processed	3.53	85.47	3.13	36.11	362.27	250.86	4.56	1.07	0.19	81.93	13.61	241.49	166.84
Icariin	crude	2.78	2182.94	7.67	2218.89	3056.92	3098.33	11.35	7.42	3.35	3613.00	1930.35	4296.51	2585.21
	processed	11.84	1706.46	7.12	2866.10	3363.19	3273.89	19.19	13.51	9.27	3605.64	2456.70	5209.31	4034.14
Astragalin	crude	294.29	23.80	688.80	76.14	10.24	30.64	695.71	730.92	356.78	32.58	56.17	18.61	62.14
	processed	237.30	7.20	668.65	38.99	6.98	25.27	606.98	679.07	305.06	24.42	24.07	9.59	97.46

**Table 5:** Factors and levels of the orthogonal array design L9 (3) ^4^ matrix.

Levels	Factors		
	Processing Heat (w)	Processed Time (min)	Weight (g)
1	200	5	5
2	400	10	10
3	600	15	15

**Table 6:** Mass spectra properties of 16 analytes.

Compounds	Precursor Ion (*m*/*z*)	Product Ion (*m*/*z*)	Frag. (V)	C.E. (V)
Magnoflorine	340.1	310.1	145	22
Neochlorogenic acid	352.9	191.0	115	10
Chlorogenic acid	353.0	191.0	90	10
Cryptochlorogenic acid	353.1	172.9	100	10
Astragalin	447.0	284.0	140	22
Quercitrin	447.2	300.1	145	20
Hyperoside	463.0	300.0	90	22
Baohuoside II	499.2	353.0	140	20
Baohuoside I	513.2	366.0	140	20
Icariside I	529.0	367.0	145	12
Epimedin B	645.1	365.6	145	30
Sagittatoside B	645.2	366.2	145	30
Epimedin C	659.2	365.7	145	30
Epimedin A	675.1	365.8	130	32
Sagittatoside A	675.2	367.0	145	32
Icariin	721.0	513.2	145	10

## Supplementary Materials

The Supplementary Materials are available online. Figures S1–S13: Appearance and microscopic features of batch 1–13 samples, respectively.
