# Supplementary material for: Comparison of the Active Compositions between Raw and Processed Epimedium from Different Species
Source: Molecules. 2018 Jul 7;23(7):1656. doi: 10.3390/molecules23071656 (PMC6099698; doi:10.3390/molecules23071656)
Supplement: Supplementary file 1 [file molecules-23-01656-s001.pdf]

molecules

MDPI

Article

Supplementary Materials: Comparison of the Active Compositions between Raw and Processed *Epimedium* from Different Species

Huamei Zhang [1], Hui Wang [1], Juan Wei, Xiaopeng Chen, Mengjie Sun, Huizi Ouyang, Jia Hao, Yanxu Chang, Zhiying Dou and Jun He *

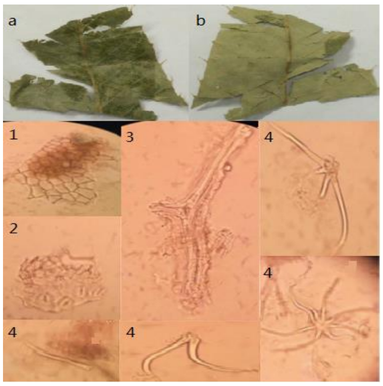

Figure S1. The appearance and microscopic features of *Epimedium* in batch 1 samples: (a) leaf upper surface; (b) leaf lower surface; (1) stem epidermis; (2) leaf lower epidermis; (3) fibers; (4) non-glandular hairs.

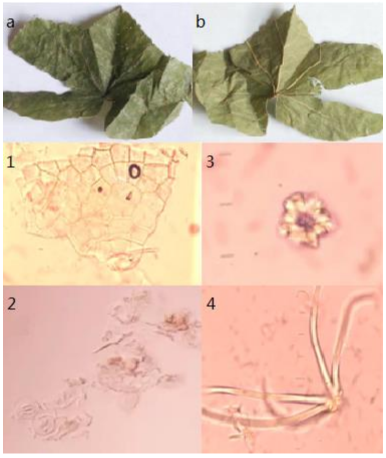

Figure S2. The appearance and microscopic features of *Epimedium* in batch 2 samples: (a) leaf upper surface; (b) leaf lower surface; (1) leaf upper epidermis; (2) leaf lower epidermis; (3) fibers; (4) non-glandular hairs.

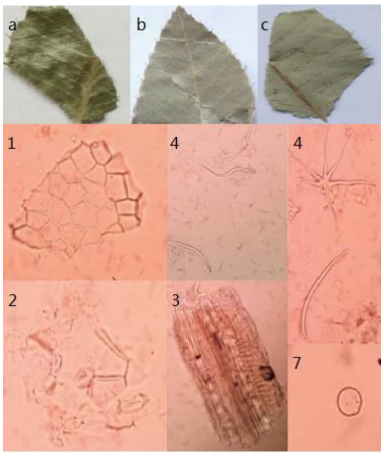

Figure S3. The appearance and microscopic features of *Epimedium* in batch 3 samples: (a) leaf upper surface; (b) tip of the leaves; (c) leaf lower surface; (1) stem epidermis; (2) leaf lower epidermis; (3) fibers; (4) non-glandular hairs; (7) oil cells.

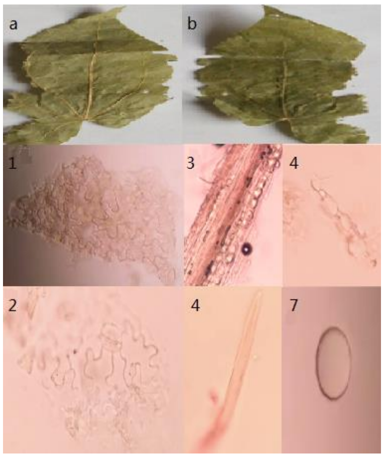

Figure S4. The appearance and microscopic features of *Epimedium* in batch 4 samples: (a) leaf upper surface; (b) leaf lower surface; (1) leaf upper epidermis; (2) leaf lower epidermis; (3) fibers; (4) non-glandular hairs; (7) oil cells.

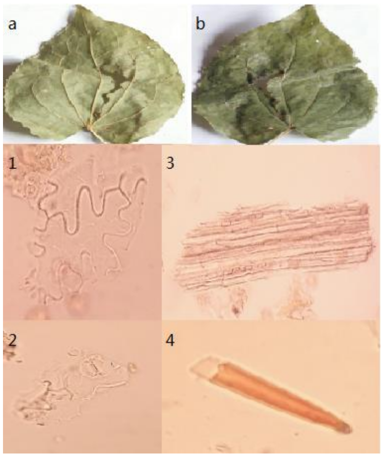

Figure S5. The appearance and microscopic features of *Epimedium* in batch 5 samples: (a) leaf upper surface; (b) leaf lower surface; (1) leaf upper epidermis; (2) leaf lower epidermis; (3) fibers; (4) non-glandular hairs.

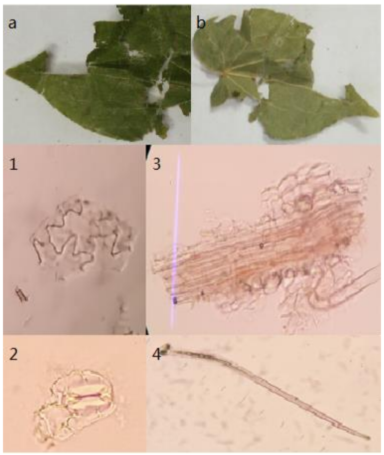

Figure S6. The appearance and microscopic features of *Epimedium* in batch 6 samples: (a) leaf upper surface; (b) leaf lower surface; (1) leaf upper epidermis; (2) leaf lower epidermis; (3) fibers; (4) non-glandular hairs.

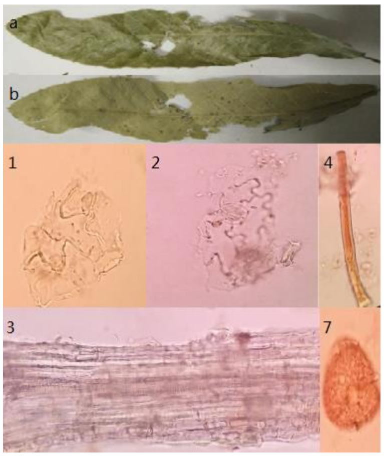

Figure S7. The appearance and microscopic features of *Epimedium* in batch 7 samples: (a) leaf upper surface; (b) leaf lower surface; (1) leaf upper epidermis; (2) leaf lower epidermis; (3) fibers; (4) non-glandular hairs; (7) oil cells.

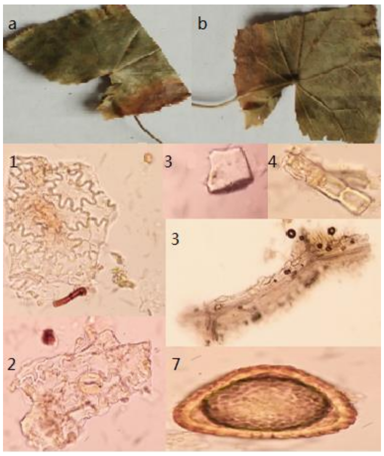

Figure S8. The appearance and microscopic features of *Epimedium* in batch 8 samples: (a) leaf upper surface; (b) leaf lower surface; (1) leaf upper epidermis; (2) leaf lower epidermis; (3) fibers; (4) non-glandular hairs; (7) oil cells.

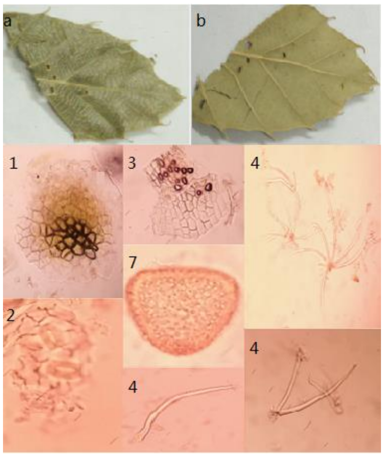

Figure S9. The appearance and microscopic features of *Epimedium* in batch 9 samples: (a) leaf upper surface; (b) leaf lower surface; (1) leaf upper epidermis; (2) leaf lower epidermis; (3) fibers; (4) non-glandular hairs; (7) oil cells.

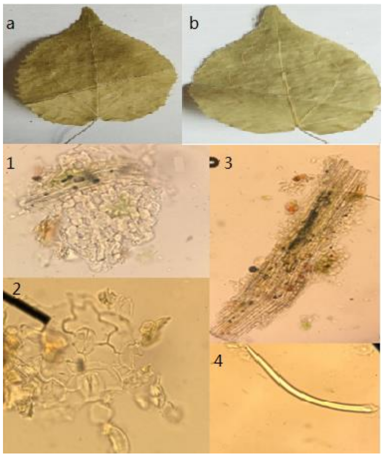

Figure S10. The appearance and microscopic features of *Epimedium* in batch 10 samples: (a) leaf upper surface; (b) leaf lower surface; (1) leaf upper epidermis; (2) leaf lower epidermis; (3) fibers; (4) non-glandular hairs.

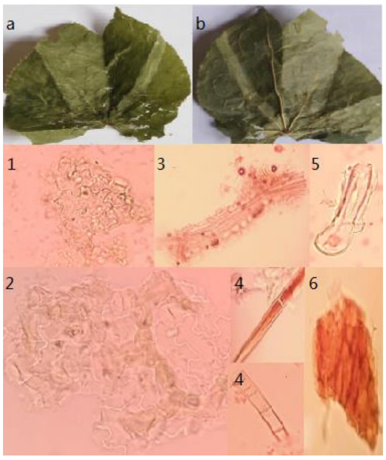

Figure S11. The appearance and microscopic features of *Epimedium* in batch 11 samples: (a) leaf upper surface; (b) leaf lower surface; (1) leaf upper epidermis; (2) leaf lower epidermis; (3) fibers; (4) non-glandular hairs; (5) glandular hairs; (6) reddish-brown content.

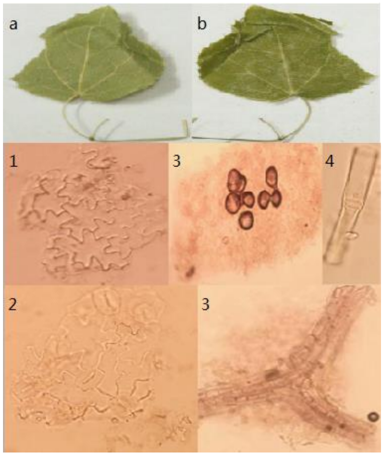

Figure S12. The appearance and microscopic features of *Epimedium* in batch 12 samples: (a) leaf upper surface; (b) leaf lower surface; (1) leaf upper epidermis; (2) leaf lower epidermis; (3) fibers; (4) non-glandular hairs.

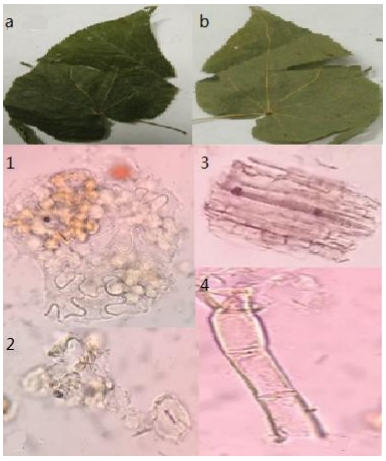

Figure S13. The appearance and microscopic features of *Epimedium* in batch 13 samples: (a) leaf upper surface; (b) leaf lower surface; (1) leaf upper epidermis; (2) leaf lower epidermis; (3) fibers; (4) non-glandular hairs.
